# Pre-pregnancy primary care and accident and emergency interactions among interpreter-users versus non-interpreter-users in England: a retrospective cohort study

**DOI:** 10.1136/bmjph-2025-003450

**Published:** 2026-08-04

**Authors:** Majel McGranahan, Nuria Sanchez Clemente, Lars Murdock, Baboucarr Njie, Yamina Boukari, Zach Welshman, Alexia Sampri, Oyinlola Oyebode, Felicity Boardman, Robert Aldridge, Neha Pathak, on behalf of the CVD-COVIDUK/COVID-IMPACT Consortium

**Affiliations:** 1 University of Warwick Medical School, Coventry, UK; 2 University College London, London, UK; 3 London School of Hygiene & Tropical Medicine, London, UK; 4 St George’s University of London, London, UK; 5 British Heart Foundation Data Science Centre, Health Data Research UK, London, UK; 6 Bennett Institute of Applied Data Science, University of Oxford, Oxford, UK; 7 British Heart Foundation Cardiovascular Epidemiology Unit, Department of Public Health and Primary Care, University of Cambridge, Cambridge, UK; 8 Victor Phillip Dahdaleh Heart and Lung Research Institute, University of Cambridge, Cambridge, UK; 9 Wolfson Institute of Population Health, Barts and The London School of Medicine and Dentistry, Queen Mary University of London, London, UK; 10 Institute for Health Metrics and Evaluation, University of Washington, Seattle, Washington, USA; 11 Central and North West London NHS Foundation Trust, London, UK; 12 University College London Hospitals NHS Foundation Trust, London, UK; 13 Health Data Research UK, London, UK

**Keywords:** Public Health, Epidemiologic Factors, Female, Health Services Accessibility

## Abstract

**Introduction:**

Non-English-speaking migrant women who need interpreters face healthcare barriers, exacerbating maternal health inequalities. Primary care-based preconception interventions can reduce preconception risk factors but require patient engagement, which has been affected by COVID-19. We aimed to identify whether women interact with general practice (GP) and accident and emergency (A&E) in the year pre-pregnancy, differences between interpreter-users and non-interpreter-users, and changes during COVID-19.

**Methods:**

English population-wide linked patient-level administrative data including primary, secondary and maternity care were used. Women with estimated pregnancy start dates from 1 March 2019 to 29 February 2024, aged 18–49 were included.

Outcomes were interactions with GP and A&E in the year pre-pregnancy (yes/no) according to interpreter-use. Outcomes were recorded according to pregnancy start date: 1 March 2019 to 29 February 2020 (pre-pregnancy-year pre-COVID-19-onset), 1 March 2020 to 28 February 2021 (pre-pregnancy-year overlapping with COVID-19-onset) or 1 March 2020 to 29 February 2024 (pre-pregnancy-year after COVID-19-onset). Logistic regression compared GP/A&E interaction pre-pregnancy among interpreter-users versus non-interpreter-users.

**Results:**

Among 2 182 280 women, 61 140 (2.8%) used interpreters. Median age was 31.0 among interpreter-users and 30.9 among non-interpreter-users. 49.7% (n=30 370) of interpreter-users were in the most deprived quintile versus 22.7% (n=480 470) of non-interpreter-users, and 62.5% (n=38 150) were of ethnic minorities (excluding white minorities) versus 22.1% (n=468 530) of non-interpreter-users.

79.5% (n=48 625) of interpreter-users interacted with their GP pre-pregnancy, versus 85.0% (n=1 802 925) of non-interpreter-users. Interpreter-use was associated with lower adjusted odds of GP interaction (aOR 0.74 (95% CI 0.72 to 0.75)), with lower odds among women whose pre-pregnancy year was post-COVID-onset.

Adjusted odds of A&E interaction in the year pre-pregnancy were lower among interpreter-users, with minimal change during COVID-19.

**Conclusions:**

Although interpreter-users were less likely to interact with GP, indicating barriers to healthcare access, most still did, making GP a promising avenue for addressing preconception health. Ensuring interpreter-need is recorded at GP registration is important. Widening inequalities since COVID-19 suggest a proactive approach is required to reduce inequalities.

**WHAT IS ALREADY KNOWN ON THIS TOPIC**

- Non-English-speaking women face healthcare barriers, and primary care-based preconception interventions can reduce preconception risk factors. However, previously, we did not know whether women using interpreters interact with general practice (GP) or accident and emergency (A&E) differently from non-interpreter-users, and whether and how interactions with GP/A&E pre-pregnancy have changed since COVID-19.

**WHAT THIS STUDY ADDS**

- This research shows that most women interact with the GP at some point in the year pre-pregnancy, but fewer interpreter-users do so, and inequalities have increased since COVID-19, suggesting a more proactive approach to preconception health may be needed. Most women do not interact with A&E in the year before getting pregnant.

**HOW THIS STUDY MIGHT AFFECT RESEARCH, PRACTICE OR POLICY**

- This study shows that clinicians in primary care are ideally placed to offer preconception health interventions but targeted interventions in multiple languages may be needed outside GP for those not accessing GP care before they get pregnant.

## Introduction

 In 2023, nearly one in three UK births were to women born abroad,^1^ many of whom require interpreters when accessing healthcare.^2^ (We have used the terms women/woman, but recognise not all pregnant or birthing people identify as women.) Maternal health inequalities exist between migrant and non-migrant women^3^ and a woman’s preconception health has a strong influence on pregnancy outcomes.^4^ A recent report into UK maternal deaths found that pre-existing conditions and inequalities must be focused on to improve outcomes.^5^

Analysis of the UK’s national Maternity Services Dataset showed that migrant women in vulnerable situations are less likely to take folic acid pre-pregnancy, more likely to have hepatitis B and diabetes preconception, and to access antenatal care late compared with UK-born women.^6^ Non-English-speaking women face additional barriers to care,^7^ with NHS England’s Improvement framework and MBRRACE-UK Perinatal Confidential Enquiry both highlighting the importance of interpreting services.^7,8^

Primary care-based preconception interventions, such as intensive or brief education, diet modification and supplementary medication, can reduce preconception risk factors.^9^ Although general practices (GPs) do not routinely provide preconception care to most women,^10^ the Clinical Knowledge Summary produced by the National Institute for Health and Care Excellence for preconception advice and management recommends that GPs discuss preconception issues with women of childbearing age during GP consultations when appropriate^11^; however, this is only possible when women interact with their GP.

A UK cross-sectional study of primary care records of 193 578 women from 2017 to 2018 found that 86.6% of women had face-to-face contact with a GP or nurse during the year before pregnancy.^12^ Although primary care appointment volumes decreased during COVID-19 lockdowns, they have since recovered to pre-pandemic levels for the general population.^13^ However, little is known about whether women using interpreters access care differently, how COVID-19 impacted GP interactions preconception and, if women interact with GPs differently preconception since COVID-19, whether any differences vary by interpreter-use.

No previous studies could be identified on preconception interventions in an accident and emergency (A&E) setting, and there is no specific guidance or policy regarding preconception care in A&E. Nonetheless, A&E could provide an opportunity to intervene to improve health preconception for those who face barriers accessing GP, including asylum seekers and refugees who do not speak English,^14^ if women attend A&E before pregnancy. This could include, for example, offering women experiencing a miscarriage support and guidance regarding planning future pregnancies, or offering contraception to those not planning pregnancy.

Although deprivation has been associated with increased A&E attendance in general,^15^ no previous study has explored A&E attendance in the year preconception, or whether there are differences in attendance between interpreter-users and non-interpreter-users, so it is unknown whether A&E could be another avenue for preconception health improvement interventions.

To develop effective opportunistic interventions to improve preconception health within GP or A&E, we need an up-to-date understanding of how women interact with GP and A&E before getting pregnant and whether this has changed with COVID-19. We also need an understanding of whether interpreter-users interact differently with GP and A&E, so that interventions can be adapted if necessary.

We aimed to describe whether women interact with GP and A&E in the year pre-pregnancy, differences between interpreter-users and non-interpreter-users, and changes before, during and after the onset of COVID-19.

## Materials and methods

We have followed the **RE**porting of studies **C**onducted using **O**bservational **R**outinely-collected **D**ata (RECORD) statement in the reporting of this study.^16^

### Study design and setting

This retrospective observational cohort study used data from an English population-based national person-level linked dataset of primary care (General Practice Extraction Service Data for Pandemic Planning and Research (GDPPR),^17^ secondary care (Hospital Episode Statistics) and maternity records (Maternity Services Data Set (MSDS)) within NHS England Secure Data Environment service for England, accessed through the British Heart Foundation Data Science Centre’s CVD-COVID-UK/COVID-IMPACT Consortium (see online supplemental file 4 for a list of consortium members). The methods for data linkage have been described in detail elsewhere.^18^

### Study population

#### Inclusion criteria

Women were included if they had singleton pregnancies recorded within MSDS with estimated pregnancy start dates between 1 March 2019 and 29 February 2024, aged 18–49 years at estimated pregnancy start date, and were alive on 1 November 2019, with a GP record at least 1 year before the estimated pregnancy start date (to ensure they were registered with the GP before their pre-pregnancy year) at one of the 98% of participating GPs in England^19^ (see online supplemental file 1 for how estimated pregnancy start date was calculated). For women with multiple pregnancies during the study period, only their first pregnancy during the study period (regardless of pregnancy outcome) was included.

#### Exclusion criteria

We excluded women who had a preceding pregnancy before the start of the study period if it was within the year pre-pregnancy of an index pregnancy (as it was not possible to tell which pregnancy GP interactions related to).

### Variables

#### Exposure

Interpreter use was defined as having codes related to interpreter use (or the need for an interpreter) recorded within a person’s GP clinical record at any time. Interpreter use within other settings (such as secondary care) was not included. NHS guidance suggests that a patient’s need for an interpreter should be recorded within their clinical notes within primary care and interpreters should be provided free of charge where needed.^20^ Use of informal interpreters (which are not recorded in clinical notes), or those with a need for interpreters but without this need recorded within clinical notes as clinical codes, were considered non-interpreter-users for the purposes of this study. We were unable to identify which languages women spoke, as some codes were limited to simply stating that an interpreter was needed (rather than in what language).

SNOMED codes describing interpreter use or need within GP (see https://github.com/BHFDSC/CCU063_03/tree/main/phenotypes) were used to identify those who had used interpreters within GP at any point since the start of their record (the earliest recorded interpreter code was from 1974) until 4 February 2025. SNOMED codes are usually added to a patient’s record when there is some kind of interaction (or when the patient registers with a GP practice) in order to record events during that interaction. We adapted and added to a pre-existing codelist related to migrant codes^21^ to create the interpreter codelist.

#### Outcomes

Outcome measures were interactions with GP (excluding prescriptions) and A&E attendances (including walk-in centres and minor injury units) in the year pre-pregnancy (yes/no), according to interpreter use (see https://digital.nhs.uk/coronavirus/gpes-data-for-pandemic-planning-and-research/guide-for-analysts-and-users-of-the-data#code-clusters-and-content for codes available within GDPPR). We also examined interactions with GP and A&E according to pre-pregnancy year relative to COVID-19 (before, overlapping with or after 1 March 2020).

The 500 most frequently occurring codes were reviewed with a practising GP to ensure they corresponded to GP interactions. Codes not considered representative of GP interactions were removed (see online supplemental file 1). See online supplemental file 2 for the 500 most frequently occurring codes after removal of those not considered representative of GP interactions.

### Bias

To understand the potential for selection bias, for individuals with interpreter codes within their GP record, we summarised when an interpreter code was first recorded in relation to their pregnancy (before the year pre-pregnancy, during the year pre-pregnancy, during pregnancy or after the recorded delivery date (or last recorded maternity interaction if delivery date unavailable)) to determine if it was possible that recorded interactions were due to interpreter-use being recorded during the pre-pregnancy year.

Variables considered to be confounders for GP and A&E interaction were individuals’ age (years), deprivation quintile (based on Index of Multiple Deprivation—an area-based relative deprivation measure), ethnic group (five categories—see table 1), geographical region (nine categories—see table 1), previous pregnancy (recorded from MSDS antenatal booking data, see online supplemental file 1 for details). These variables were adjusted for in regression analysis (see below).

**Table 1:** Demographic characteristics of study population (women with estimated pregnancy start dates between 1 March 2019 and 29 February 2024, aged 18–49)

Characteristic	Overall	Interpreter-users	Non-interpreter-users
	(N=2 182 280)	(N=61 140)	(N=2 121 140)
Age (years)			
Mean (SD)	30.7 (5.51)	30.9 (5.84)	30.7 (5.50)
Median (IQR)	30.9 (23.3, 38.5)	31.0 (22.5, 39.4)	30.9 (23.3, 38.4)
Ethnic group			
White	1 673 735 (76.7%)	22 930 (37.5%)	1 650 805 (77.8%)
Black, Black British, Caribbean or African	104 095 (4.8%)	6705 (11.0%)	97 390 (4.6%)
Asian or Asian British	286 355 (13.1%)	20 780 (34.0%)	265 575 (12.5%)
Mixed or multiple ethnic groups	57 290 (2.6%)	2380 (3.9%)	54 910 (2.6%)
Other ethnic group	58 935 (2.7%)	8280 (13.5%)	50 655 (2.4%)
Missing	1870 (0.1%)	60 (0.1%)	1810 (0.1%)
Deprivation quintile (Index of Multiple Deprivation)			
1 (most deprived)	510 840 (23.4%)	30 370 (49.7%)	480 470 (22.7%)
2	457 555 (21.0%)	16 480 (27.0%)	441 075 (20.8%)
3	426 940 (19.6%)	7690 (12.6%)	419 250 (19.8%)
4	406 720 (18.6%)	4165 (6.8%)	402 560 (19.0%)
5 (least deprived)	378 015 (17.3%)	2420 (4.0%)	375 600 (17.7%)
Missing	2205 (0.1%)	15 (0.0%)	2190 (0.1%)
Previous pregnancy			
No	790 275 (36.2%)	13 150 (21.5%)	777 125 (36.6%)
Yes	1 213 280 (55.6 %)	42 195 (69.0%)	1 171 090 (55.2%)
Missing	178 725 (8.2%)	5795 (9.5%)	172 930 (8.2%)
Region			
London	359 525 (16 %)	19 650 (32.1%)	339 875 (16.0%)
South East	356 430 (16.5%)	3135 (5.1%)	353 290 (16.7%)
South West	195 465 (9.0%)	1460 (2.4%)	194 010 (9.1%)
East of England	248 910 (11.4%)	4755 (7.8%)	244 155 (11.5%)
East Midlands	184 570 (8.5%)	5725 (9.4%)	178 840 (8.4%)
West Midlands	234 740 (10.8%)	5935 (9.7%)	228 805 (10.8%)
Yorkshire and The Humber	212 920 (9.8%)	11 005 (18.0%)	201 910 (9.5%)
North East	98 405 (4.5%)	2225 (3.6%)	96 180 (4.5%)
North West	289 110 (13.2%)	7225 (11.8%)	281 890 (13.3%)
Missing	2205 (0.1%)	15 (0.0%)	2190 (0.1%)

Age, deprivation quintile, geographical region and ethnicity were recorded according to BHF data science centre methods described elsewhere^22^ (code available from https://github.com/BHFDSC/hds_curated_assets/blob/main/D07-demographics.py). Where age was unavailable from the aforementioned BHF data science centre methods, the age derived from MSDS booking data was used.

### Study size

MSDS coverage was more limited earlier on during the study,^23^ meaning a relatively smaller cohort of women were included in earlier years; however, the cohort was still representative of pregnant women in England and the study was sufficiently powered given the large sample size throughout the study period.

### Statistical methods

Two logistic regression models were developed to quantify the association between interpreter-use, the key exposure of interest, and (a) GP interactions in the year pre-pregnancy (yes/no) and (b) A&E interactions in the year pre-pregnancy (yes/no). Adjustment for confounding factors was based on factors available in the dataset that are known to be associated with healthcare attendance and likely to be associated with interpreter use. Forced entry model fitting was used (covariates were added to the regression model simultaneously). Confounding factors adjusted for included mother’s age (years), deprivation quintile, ethnic group (five categories, see table 1), geographical region (nine categories, see table 1) and previous pregnancy (yes/no—according to MSDS antenatal booking data). For logistic regression analyses, individuals with missing data for any covariate were excluded from the analyses.

Logistic regression analyses were undertaken for the whole study period and according to whether individual’s pre-pregnancy year was before, overlapping with or after 1 March 2020.

Because of the complex relationship between ethnicity and migration,^24^ and therefore interpreter use, sensitivity analysis explored the contribution of adjustment for ethnic group by including a multivariable model that did not adjust for ethnic group.

### Data cleaning

To comply with NHS England statistical disclosure control guidance, counts were rounded to the nearest five and cells smaller than ten were suppressed.

Analysis was undertaken following a prespecified protocol using R V.4.1.3, and data curation was undertaken using Pyspark V.3.3.0 within Databricks. The study protocol and code used for the data curation and analysis are available online (https://github.com/BHFDSC/CCU063_03).

### Patient and public involvement

A community advisory group consisting of migrant women and healthcare professionals was involved in developing the research proposal and provided feedback on the research questions and study design. The community advisory group directly contributed to the decision to include both GP and A&E data, as these were felt particularly relevant to this group of women. The group also offered insights into interpretation of results.

## Results

**Figure 1:**
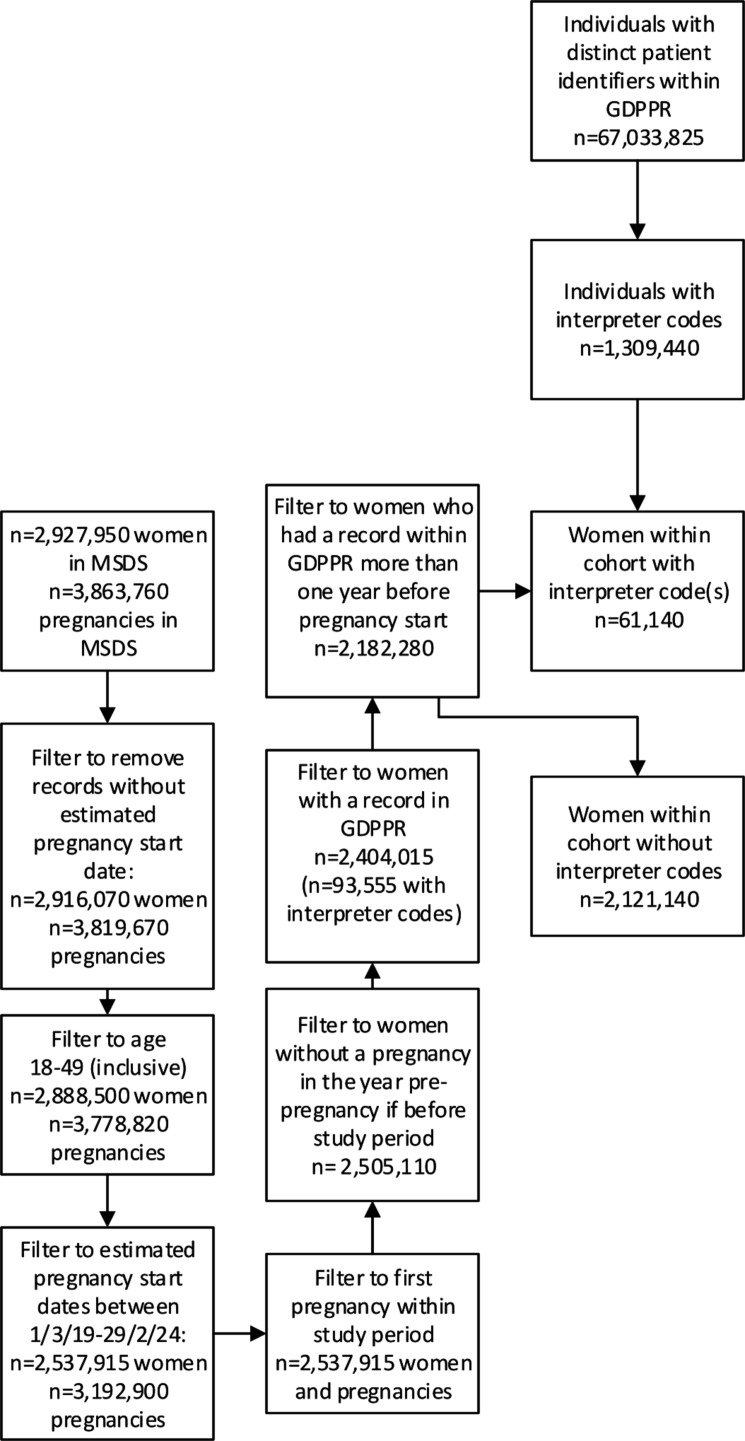
Number of women in the study including exclusions. GDPPR, General Practice Extraction Service Data for Pandemic Planning and Research; MSDS, Maternity Services Data Set.

As shown in table 1, there were 2 182 280 women with singleton pregnancies recorded within MSDS with estimated pregnancy start dates between 1 March 2019 and 29 February 2024, aged 18–49 years at estimated pregnancy start date. 61 140 (2.8%) had a record of interpreter-use and 2 121 140 (97.2%) did not. The median age of the study population was 31.0 among interpreter-users and 30.9 among non-interpreter-users. Among interpreter-users, 37.5% were of white ethnicity compared with 77.8% of non-interpreter-users. Half (49.7%) of interpreter-users were in the most deprived quintile, compared with 22.7% of non-interpreter-users. Figure 1 shows the reasons for exclusions.

As shown in table 2, among both interpreter-users and non-interpreter-users, GP interactions during the year pre-pregnancy were lowest for those whose year pre-pregnancy overlapped with the start of COVID-19 (1 March 2020), and highest for those whose year pre-pregnancy was after the start of COVID-19. GP interactions were lower among interpreter-users than non-interpreter-users both before, during and after the onset of COVID-19.

**Table 2:** Proportion of individuals with GP and A&E interactions in the year pre-pregnancy among interpreter-users and non-interpreter-users according to period relative to the onset of COVID-19 (defined as 1 March 2020)

	Overall	Interpreter-users	Non-interpreter-users
GP interactions in the year pre-pregnancy			
Whole study period	n*=*2 182 280	n=61 140	n=2 121 140
One or more	1 851 550 (84.8%)	48 625 (79.5%)	1 802 925 (85.0%)
Pre-pregnancy year before 1 March 2020	N=5 47 450	N=15 275	N=5 32 175
One or more	447 450 (81.7%)	12 280 (80.4%)	435 165 (81.8%)
Pre-pregnancy year overlaps with 1 March 2020	N=5 18 810	N=14 160	N=5 04 650
One or more	415 675 (80.1%)	10 565 (74.6%)	405 110 (80.3%)
Pre-pregnancy year after 1 March 2020	N=1 116 020	N=31 700	N=1 084 320
One or more	988 430 (88.6%)	25 780 (81.3%)	962 650 (88.8%)
A&E attendance in the year pre-pregnancy			
Whole study period	n*=*2 182 280	n=61 140	n=2 121 140
One or more	563 675 (25.8%)	17 240 (28.2%)	546 440 (25.8%)
Pre-pregnancy year before 1 March 2020	N=5 47 450	N=15 275	N=5 32 175
One or more	149 470 (27.3%)	4465 (29.2%)	145 000 (27.2%)
Pre-pregnancy year overlaps with 1 March 2020	N=5 18 810	N=14 160	N=5 04 650
One or more	121 480 (23.4%)	3610 (25.5%)	117 870 (23.4%)
Pre-pregnancy year after 1 March 2020	N=1 116 020	N=31 700	N=1 084 320
One or more	292 730 (26.2%)	9160 (28.9%)	283 570 (26.2%)

Individuals consist of women with estimated pregnancy start dates between 1 March 2019 and 29 February 2024, aged 18–49.

A&E, accident and emergency; GP, general practice.

As shown in table 3, the adjusted odds of interaction with GP in the year pre-pregnancy were 0.74 (95% CI 0.72 to 0.75) times lower among interpreter-users versus non-interpreter-users during the study period overall. Before COVID-19, the adjusted odds of GP interaction among interpreter-users compared with non-interpreter-users was 0.92 (95% CI 0.88 to 0.96); this lowered to 0.63 (95% CI 0.61 to 0.65) for those with pre-pregnancy years after the onset of COVID-19.

**Table 3:** Logistic regression models showing odds of GP interaction and A&E attendance among interpreter-users, with non-interpreter-users as the reference category

Outcome	Unadjusted OR (95% CI)	Adjusted OR (95% CI)
GP interaction in year pre-pregnancy for the whole study population		
	0.67 (0.66 to 0.69)*	0.74 (0.72 to 0.75)*
GP interaction in year pre-pregnancy for women whose pre-pregnancy year was before 1 March 2020		
	0.90 (0.86 to 0.94)*	0.92 (0.88 to 0.96)*
GP interaction in year pre-pregnancy for women whose pre-pregnancy year overlapped with 1 March 2020		
	0.71 (0.69 to 0.74)*	0.76 (0.73 to 0.79)*
GP interaction in year pre-pregnancy for women whose pre-pregnancy year was after 1 March 2020		
	0.55 (0.53 to 0.56)*	0.63 (0.61 to 0.65)*
A&E attendance in the year pre-pregnancy for the whole study population		
	1.12 (1.10 to 1.14)*	0.96 (0.95 to 0.98)*
A&E interaction in year pre-pregnancy for women whose pre-pregnancy year was before 1 March 2020		
	1.08 (1.04 to 1.12)*	0.93 (0.90 to 0.97)
A&E interaction in year pre-pregnancy for women whose pre-pregnancy year overlapped with 1 March 2020		
	1.11 (1.06 to 1.15)*	0.95 (0.91 to 0.99)
A&E interaction in year pre-pregnancy for women whose pre-pregnancy year was after 1 March 2020		
	1.14 (1.11 to 1.17)*	0.98 (0.95 to 1.00)

Individuals consist of women with estimated pregnancy start dates between 1 March 2019 and 29 February 2024, aged 18–49.

Fully adjusted models adjust for mother’s age (years), ethnic group, geographical region, deprivation quintile and previous pregnancy (binary yes/no). COVID-19 onset defined as 1 March 2020. See online supplemental file 3 for full regression models.

* p<0.001*.*

A&E, accident and emergency; GP, general practice.

Overall, A&E interactions were lowest for those whose pre-pregnancy year overlapped with the start of COVID-19 (1 March 2020), and highest for those whose pre-pregnancy year was before the start of COVID-19. A higher proportion of interpreter-users accessed A&E in the year pre-pregnancy than non-interpreter-users during all COVID-19-related time periods.

The unadjusted odds of interaction with A&E in the year pre-pregnancy were 1.12 (95% CI 1.10 to 1.14) higher among interpreter-users compared with non-interpreter-users, but 0.96 (95% CI 0.95 to 0.98) times lower among interpreter-users when adjusted for confounding factors. The adjusted odds of A&E interactions did not change substantially between individuals with pre-pregnancy years occurring pre-COVID-19, during and post-COVID-19 onset.

The addition/removal of ethnic group as a covariate did not substantially change odds of GP or A&E interactions (online supplemental file 3), indicating there was no independent contribution of ethnicity on GP/A&E interactions.

See online supplemental file 3 for full regression models including all covariates.

Within the study population, the majority of the interpreter-user group (44 245, 72.4%) had their first interpreter code recorded before the year pre-pregnancy (table 4); 5.2% (n=3170) had their first interpreter code recorded during the year pre-pregnancy.

**Table 4:** Period of interpreter recording for study participants who have interpreter recorded. Individuals consist of women with estimated pregnancy start dates between 1 March 2019 and 29 February 2024, aged 18–49

Period interpreter first recorded within GP record	Women included in the study population Number (%)
Prior to pre-pregnancy year	44 245 (72.4)
During year pre-pregnancy	3170 (5.2)
After pregnancy start (during pregnancy)	4740 (7.8)
After delivery*	8985 (14.7)

* For those without a recorded delivery date within the Maternity Services Data Set (MSDS), the last recorded entry for the pregnancy within MSDS was used as a proxy for delivery date.

GP, general practice.

## Discussion

### Key results and interpretation

We have found that the majority of women interact with GP in the year before getting pregnant, and this proportion increased from 85.0% of non-interpreter-users and 79.5% of interpreter-users for pregnancy start dates pre-COVID-19 to 88.8% of non-interpreter-users and 81.3% of interpreter-users for pregnancy start dates post-COVID-19 onset (1 March 2020). Women using interpreters had lower odds of interacting with GP in the year pre-pregnancy compared with non-interpreter-users, and the odds of interaction with GP reduced progressively from pre-COVID-19 (pregnancy start dates occurring before 1 March 2020) to post-COVID-19 (pregnancy start dates occurring after 28 February 2021), suggesting that inequalities between interpreter-users and non-interpreter-users in primary care attendance have increased.

The majority of women included in the study had their first interpreter use recorded before the year pre-pregnancy; but 27.6% did not. This suggests that targeted information about preconception health in languages appropriate for women registered at a practice is challenging because it is not necessarily possible to ascertain which languages women speak until after they have conceived. Ensuring that language preference and need for interpreter are recorded at GP registration is important to ensure that health interventions can be provided in appropriate languages and format.

Most women did not interact with A&E in the year pre-pregnancy, so delivering preconception interventions within A&E would have limited reach compared with primary care. In any case, A&E may be less suited to preconception interventions compared with primary care, given the lack of continuity of care and the focus on urgent versus preventative care.

### Comparison with existing literature

This study builds on a previous study using 2017–2018 data exploring pre-pregnancy care in GP in England which found that 86.6% of women had contact with a GP or nurse in the year pre-pregnancy.^12^ Our study found that, since COVID-19, this proportion has increased in the general population, indicating that interventions to improve preconception health within primary care could have more reach than ever in the general population, but that women using interpreters were less likely to interact with the GP, indicating barriers to primary care access.

A previous study exploring migrants’ primary care utilisation before and during COVID-19 in England found that migrants used primary care less than non-migrants, and this difference increased in the first year of the pandemic.^25^ Interpreter-users are a subset of migrants that face additional barriers to accessing and using healthcare.^26^ Although our study did not examine migrants in particular, there is likely to be significant overlap between interpreter-users and migrants, indicating that both populations may need targeted preconception support outside of primary care.

Rates of A&E attendance were similar in our study compared with national averages (considering that women of childbearing age have higher rates of attendance compared with males).^15^ Although no previous study could be identified which has explored the association between interpreter use and A&E attendance, deprivation is associated with increased attendance^15^ which may explain why odds of A&E attendance in the year preconception were higher among interpreter-users in unadjusted logistic regression models, but not in adjusted models.

There are complex reasons for healthcare attendance,^27^ and it is likely that it is not simply the use of an interpreter per se that results in fewer interactions with primary care. The ‘requiring interpreter’ variable is likely to be acting as a proxy for other unrecorded variables; for example, migration status (non-migrant vs migrant), years since migration (for migrants, due to the ‘healthy migrant effect’^28^), cultural beliefs (eg, a conscious pre-pregnancy period not being relatable),^29^ and healthcare barriers, for example transport and distrust of male doctors or interpreters.^29^

Data from the Migration Observatory indicates that 99% of people born in the UK consider English as a first language.^30^ Therefore, there is a very small chance that individuals requiring an interpreter were UK-born: interpreter-users are predominantly migrants,^30^ with complex barriers to healthcare beyond language.^31^ This is particularly pertinent given that nearly one in three births in England and Wales were to women born outside the UK in 2023,^1^ and the recent MBRRACE-UK report highlighting the challenging healthcare environment faced by women with language barriers within maternity.^7^

### Strengths and limitations

This is the first study to explore interactions with GP and A&E in the preconception period since the COVID-19 pandemic, and the first study to explore interactions among women using interpreters. The national linked dataset we used includes primary care, Hospital Episode Statistics and MSDS, providing a unique opportunity to identify women from their first antenatal booking appointment (Hospital Episode Statistics Maternity records do not routinely include antenatal appointments, so largely capture pregnancies that involve a hospital delivery). This means that we were able to include some pregnant women who did not have a successful pregnancy, which previous related studies have not.^12^ This means that women who had pregnancy losses after their initial midwife appointment were included; miscarriages occur in 15.3% of recognised pregnancies and these pregnancies may have different preconception risk factors^32^ so are important to consider when undertaking preconception health research.

A real strength of this study is that there were very few missing data for ethnic group (0.1%) because ethnicity could be drawn from any of several data sources^22^; this is particularly important given the well-established evidence on ethnicity and maternal health outcomes.^5^ However, it was not possible to differentiate between ‘White British’ and ‘White other’ ethnic groups, and the study population is limited to over-18s so implications for teenage pregnancies, which are more common in deprived areas,^33^ cannot be inferred.

We identified that inequalities in GP interactions increased progressively over time across pandemic periods (pre, during, post). Each of these time periods will be heterogeneous with regards to COVID-19 restrictions. In particular, the post-COVID-19-onset group includes some women who will have experienced COVID-19 control measures (such as lockdowns) for some of their pre-pregnancy year which may have influenced GP interactions. However, this group, as a whole, is likely to have experienced far fewer COVID-19 related restrictions compared with the group with pre-pregnancy years overlapping with COVID-19 onset so this still provides a valuable indication of the change in interactions over this time period.

For women with interpreter codes, there is a possibility of self-selection bias because we defined our cases based on interpreter code, which could involve an additional interaction with GP if recorded on a different date to GP registration. Between 2008 and 2011, there was an incentive for GPs to record language use under the Quality Outcomes Framework,^21^ meaning women registered during this time may be more likely to have interpreter-use recorded. People who need an interpreter, but who did not visit the GP so this need was not recorded, or used an informal interpreter (not recommended by NHS guidelines^20^), are included in the ‘non-interpreter-users’ group, and are therefore contributing to an under-estimate of interactions with primary care in the non-interpreter-users group due to this misclassification. However, it is highly likely that the dominant group is those who do not require an interpreter (since 99% of people born in the UK consider English their first language^30^). We still observed that interpreter-users were less likely to interact with GP in the year pre-pregnancy compared with non-interpreter-users, but this may have underestimated our effect size which would mean inequalities are more substantial than they appear.

Not all GP codes were available within the data due to the nature of GDPPR having been developed to study COVID-19 and cardiovascular disease^18^; however, the dataset has been used previously to estimate healthcare use^34^ and frequency of GP interactions was similar to those found elsewhere.^12^

Many women using interpreters were excluded from the study population because they had not interacted with the GP before the year preconception so did not meet the inclusion criteria (n=32 415, 34.6% of interpreter users pre-exclusions compared with n=189 230, 8.2% of non-interpreter users pre-exclusions). We therefore selected people for inclusion in the study who have been accessing services in some way, again potentially underestimating inequalities since a larger proportion of interpreter-users were excluded for this reason than non-interpreter-users.

Finally, because we only included the first pregnancy per woman within the study period, it may be assumed that women in later cohorts would be younger; however, the median age of the population over the three time periods did not substantially change (median age 30.7 for those whose pregnancy start date was before 1 March 2020, 31.0 for those whose pregnancy start date was between 1 March 2020 and 28 February 2021, and 30.9 for those whose pregnancy start date was after 28 February 2021).

### Implications for research and practice

This study highlights the potential for primary care to improve preconception health given the high proportion of women interacting with GP pre-pregnancy.^31^ As detailed in the Lancet Commission on Migration and Health framework for migrants’ access to health and social protection, access to healthcare is complex and consideration of other barriers to access must be considered alongside language.^31^ For example, fear of deportation, lack of awareness of the NHS, transport difficulties and perception of discrimination can all act as barriers to access.^14,31,35^ Proactively reaching out to women who are less likely to interact with primary care, such as interpreter-users and other migrants,^25^ could offer further opportunities for intervention. During patient interactions, GPs could then provide brief interventions to improve preconception health, for example through asking women of reproductive age about their pregnancy intentions,^36^ provide brief advice where needed (eg, through the use of motivational interviews), or identify those who require further input outside of the consultation (eg, referral to secondary care to optimise a chronic health condition before pregnancy).^11,36^

The Women’s Health Strategy for England (currently being updated) has a focus on inequalities and preconception health^37^ and presents an opportunity for primary care to play a key role in reducing preconception health inequalities. However, the move to digitalisation as part of the NHS Ten Year Health Plan 2025^38^ could exacerbate the inequalities in GP interactions identified in this study and others^25^ if translated digital resources are unavailable, language requirements are not recorded within GP records and options for those who are not digitally literate unavailable. It will be necessary to continue to monitor GP interactions as digitalisation of primary care increases to ensure that those who face language (and other) barriers are not further excluded.

We have shown that interpreter-users are less likely to interact with the GP in the year before getting pregnant than non-interpreter-users. Nonetheless, the majority of women in England interacted with the GP at some point in the year pre-pregnancy, making primary care a promising avenue for addressing preconception health. To address the widening inequalities in accessing primary care in the year preconception, GP practices could actively reach out to people on their lists to obtain information about interpretation needs and pregnancy intentions (where appropriate) to create opportunities to interact. Interventions must be adapted to consider the heterogeneity of the population. Given the substantial proportion of interpreter-users who had not interacted with the GP at any time before their pre-pregnancy year (so were unlikely to be registered), we must also ensure that interventions to improve preconception health are available outside of GP in multiple languages for those who need them, including through community groups, health visitors and other routes of access.

## Supplementary material

10.1136/bmjph-2025-003450 online supplemental file 1

10.1136/bmjph-2025-003450 online supplemental file 2

10.1136/bmjph-2025-003450 online supplemental file 3

10.1136/bmjph-2025-003450 online supplemental file 4

## Data Availability

Data may be obtained from a third party and are not publicly available.

## References

[1] Births by parents’ country of birth, England and Wales. Office for National Statistics [Internet]. https://www.ons.gov.uk/peoplepopulationandcommunity/birthsdeathsandmarriages/livebirths/bulletins/parentscountryofbirthenglandandwales/2023.

[2] MacLellan J, McNiven A, Kenyon S (2024). Provision of interpreting support for cross-cultural communication in UK maternity services: a freedom of information request. Int J Nurs Stud Adv.

[3] Heslehurst N, Brown H, Pemu A (2018). Perinatal health outcomes and care among asylum seekers and refugees: a systematic review of systematic reviews. BMC Med.

[4] Daly M, Kipping RR, Tinner LE (2022). Preconception exposures and adverse pregnancy, birth and postpartum outcomes: umbrella review of systematic reviews. Paediatr Perinat Epidemiol.

[5] Felker A, Patel R, Kotnis R (2025). Improving mothers’ care - lessons learned to inform maternity care from the UK and Ireland confidential enquiries into maternal deaths and morbidity 2021-23.

[6] McGranahan M, Augarde E, Schoenaker D (2024). Preconception health among migrant women in England: a cross-sectional analysis of maternity services data 2018-2019. J Migr Health.

[7] Perinatal confidential enquiry, MBRRACE-UK The care of recent migrant women with language barriers who have experienced a stillbirth or neonatal death. [internet]. https://timms.le.ac.uk/mbrrace-uk-perinatal-mortality/confidential-enquiries/confidential-enquiry-migrant-women.html.

[8] NHS England Improvement framework: community language translation and interpreting services [Internet].

[9] Withanage NN, Botfield JR, Srinivasan S (2022). Effectiveness of preconception interventions in primary care: a systematic review. Br J Gen Pract.

[10] Schoenaker D, Connolly A, Stephenson J (2022). Preconception care in primary care: supporting patients to have healthier pregnancies and babies. Br J Gen Pract.

[11] NICE Pre-conception – advice and management [internet]. https://cks.nice.org.uk/topics/pre-conception-advice-management/.

[12] Li Y, Kurinczuk JJ, Alderdice F (2025). Pre-pregnancy care in general practice in England: cross-sectional observational study using administrative routine health data. BMC Public Health.

[13] Zhao T, Meacock R, Sutton M (2025). Drivers of primary care appointment volumes before and after the COVID-19 pandemic: a longitudinal study. BMC Health Serv Res.

[14] Kang C, Tomkow L, Farrington R (2019). Access to primary health care for asylum seekers and refugees: a qualitative study of service user experiences in the UK. Br J Gen Pract.

[15] Office for National Statistics Inequalities in accident and emergency department attendance, England [Internet]. https://cy.ons.gov.uk/peoplepopulationandcommunity/healthandsocialcare/healthcaresystem/articles/inequalitiesinaccidentandemergencydepartmentattendanceengland/march2021tomarch2022.

[16] Benchimol EI, Smeeth L, Guttmann A (2015). The REporting of studies Conducted using Observational Routinely-collected health Data (RECORD) statement. *PLoS Med*.

[17] NHS England Digital GPES data for pandemic planning and research (COVID-19) (GDPPR) [Internet]. https://digital.nhs.uk/coronavirus/gpes-data-for-pandemic-planning-and-research.

[18] Wood A, Denholm R, Hollings S (2021). Linked electronic health records for research on a nationwide cohort of more than 54 million people in England: data resource. BMJ.

[19] GDPPR | BHF DSC HDS documentation [Internet]. https://bhfdsc.github.io/documentation/docs/dataset_insights/gdppr.

[20] NHS England (2018). Guidance for commissioners: interpreting and translation services in primary care [internet]. https://www.england.nhs.uk/publication/guidance-for-commissioners-interpreting-and-translation-services-in-primary-care/.

[21] Pathak N, Zhang CX, Boukari Y (2021). Development and validation of a primary care electronic health record phenotype to study migration and health in the UK. Int J Environ Res Public Health.

[22] Methodology [Internet] BHF DSC HDS documentation. https://bhfdsc.github.io/documentation/curated_assets/kpcs/methodology.

[23] NHS England Digital NHS maternity statistics, England, 2023–24 [Internet]. https://digital.nhs.uk/data-and-information/publications/statistical/nhs-maternity-statistics/2023-24.

[24] Vissandjee B, Desmeules M, Cao Z (2004). Integrating ethnicity and migration as determinants of Canadian women’s health. BMC Womens Health.

[25] Zhang CX, Boukari Y, Pathak N (2022). Migrants’ primary care utilisation before and during the COVID-19 pandemic in England: an interrupted time series analysis. Lancet Reg Health Eur.

[26] Pandey M, Maina RG, Amoyaw J (2021). Impacts of English language proficiency on healthcare access, use, and outcomes among immigrants: a qualitative study. BMC Health Serv Res.

[27] Thaddeus S, Maine D (1994). Too far to walk: maternal mortality in context. Soc Sci Med.

[28] Spallek J, Zeeb H, Razum O (2011). What do we have to know from migrants’ past exposures to understand their health status? A life course approach. Emerg Themes Epidemiol.

[29] McGranahan M, Boardman F, Ahmed S (2025). Opportunities, needs and barriers to preconception health and access to preferred contraception: a qualitative study of underserved migrant women. Authorea Preprints.

[30] Migration Observatory English language use and proficiency of migrants in the UK [Internet]. https://migrationobservatory.ox.ac.uk/resources/briefings/english-language-use-and-proficiency-of-migrants-in-the-uk/.

[31] Abubakar I, Aldridge RW, Devakumar D (2018). The UCL–Lancet Commission on Migration and Health: the health of a world on the move. The Lancet.

[32] Quenby S, Gallos ID, Dhillon-Smith RK (2021). Miscarriage matters: the epidemiological, physical, psychological, and economic costs of early pregnancy loss. The Lancet.

[33] Office for National Statistics Conceptions in England and Wales [Internet]. https://www.ons.gov.uk/peoplepopulationandcommunity/birthsdeathsandmarriages/conceptionandfertilityrates/bulletins/conceptionstatistics/2018#conceptions-by-index-of-multiple-deprivation.

[34] Mu Y, Dashtban A, Mizani MA (2024). Healthcare utilisation of 282,080 individuals with long COVID over two years: a multiple matched control, longitudinal cohort analysis. J R Soc Med.

[35] Khanom A, Alanazy W, Couzens L (2021). Asylum seekers’ and refugees’ experiences of accessing health care: a qualitative study. BJGP Open.

[36] Fullerton DK (2024). Pre-conception counselling. InnovAiT: Education and Inspiration for General Practice.

[37] GOV.UK Women’s health strategy for England [internet]. https://www.gov.uk/government/publications/womens-health-strategy-for-england/womens-health-strategy-for-england.

[38] (2025). Fit for the future: 10 year health plan for England [Internet]. https://www.gov.uk/government/publications/10-year-health-plan-for-england-fit-for-the-future.

