# Supplementary material for: Pre-pregnancy primary care and accident and emergency interactions among interpreter-users versus non-interpreter-users in England: a retrospective cohort study
Source: BMJ Public Health. 2026 Aug 4;4(3):e003450. doi: 10.1136/bmjph-2025-003450 (PMC13448527; doi:10.1136/bmjph-2025-003450)
Supplement: Supplementary file 1 [file bmjph-4-3-s004.pdf]

CVD-COVID-UK / COVID-IMPACT Consortium Members (11/03/2026)

British Heart Foundation Data Science Centre
Led by Health Data Research UK

Institution	Member Name
**Barts Health NHS Trust**	Alastair Proudfoot
**Barts Health NHS Trust**	Andrew Constantine
**Barts Health NHS Trust**	Dan Jones
**Barts Health NHS Trust**	Krishnaraj Rathod
**Barts Health NHS Trust**	Nida Ahmed
**Barts Health NHS Trust**	Richard Fitzgerald
**British Heart Foundation**	Dan O'Connell
**British Heart Foundation**	Rony Arafin
**British Heart Foundation**	Sonya Babu-Narayan
**British Heart Foundation**	Zainab Karim
**Cancer Research UK**	Jon Shelton
**Cancer Research UK**	Martina Slapkova
**Cancer Research UK**	Rosie Hinchliffe
**Cancer Research UK**	Shane Johnson
**Cardiff Metropolitan University**	Renin Toms
**Cardiff University**	Julia Townson
**European Bioinformatics Institute**	Ewan Birney
**European Bioinformatics Institute**	Moritz Gerstung
**European Bioinformatics Institute**	Tomas Fitzgerald
**Great Ormond Street Hospital**	Katherine Brown
**Guy's and St Thomas' NHS Foundation Trust**	Benjamin Zuckerman
**Guy's and St Thomas' NHS Foundation Trust**	Ernest Wong
**Guy's and St Thomas' NHS Foundation Trust**	Lily Benton
**Guy's and St Thomas' NHS Foundation Trust**	Tasanee Braithwaite
**Health Data Research UK**	Alexis Webb
**Health Data Research UK/BHF Data Science Centre**	Anna Stevenson
**Health Data Research UK/BHF Data Science Centre**	Beth Friel
**Health Data Research UK/BHF Data Science Centre**	Fionna Chalmers
**Health Data Research UK/BHF Data Science Centre**	Jadene Lewis
**Health Data Research UK/BHF Data Science Centre**	Jessica Farrow

Institution	Member Name
**Health Data Research UK/BHF Data Science Centre**	John Nolan
**Health Data Research UK/BHF Data Science Centre**	Lars Murdock
**Health Data Research UK/BHF Data Science Centre**	Laura Sherlock
**Health Data Research UK/BHF Data Science Centre**	Lynn Morrice
**Health Data Research UK/BHF Data Science Centre**	Melissa Webb
**Health Data Research UK/BHF Data Science Centre**	Ross Forsyth
**Health Data Research UK/BHF Data Science Centre**	Sandy Wibrew
**Health Data Research UK/BHF Data Science Centre**	Steffen Petersen
**Health Data Research UK/BHF Data Science Centre**	Thomas Bolton
**Health Data Research UK/BHF Data Science Centre**	Zach Welshman
**Healthcare Quality Improvement Partnership**	Caroline Rogers
**Heidelberg University**	Petr Andriushchenko
**Hull York Medical School**	Donna Wakefield
**Imperial College London**	Ahmed Salih
**Imperial College London**	Alun Davies
**Imperial College London**	Arunashis Sau
**Imperial College London**	Costas Kallis
**Imperial College London**	Francesca Cavallo
**Imperial College London**	Fu Siong Ng
**Imperial College London**	Hannah Whittaker
**Imperial College London**	Ioanna Tzoulaki
**Imperial College London**	Jennifer Quint
**Imperial College London**	Juliette Unwin
**Imperial College London**	Libor Pastika
**Imperial College London**	Petter Brodin
**Imperial College London**	Philip Stone
**Imperial College London**	Safa Salim
**Imperial College London**	Sarah Cook

Institution	Member Name
**Imperial College London**	Sarah Onida
**Imperial College London (LCP)**	Alistair Marsland
**Imperial College London (LCP)**	Andrew Thompson
**Imperial College London (LCP)**	Sara Holloway
**Imperial College London (LCP)**	Thomas Porter
**INSIGHT**	Alastair Denniston
**Keele University**	Mamas Mamas
**King's College London**	Abdel Douiri
**King's College London**	Adejoke Oluyase
**King's College London**	Ajay Shah
**King's College London**	Alexandru Dregan
**King's College London**	Amy Ronaldson
**King's College London**	Anna Bone
**King's College London**	Antonio Cannata
**King's College London**	Ben Bray
**King's College London**	Charles Wolfe
**King's College London**	Daniel Bromage
**King's College London**	Dominic Oliver
**King's College London**	Elena Nikiphorou
**King's College London**	Emeka Chukwusa
**King's College London**	Emel Yorganci
**King's College London**	Gareth Williams
**King's College London**	Gayan Perera
**King's College London**	Harry Watson
**King's College London**	Iain Marshall
**King's College London**	India Tunnard
**King's College London**	Irene Higginson
**King's College London**	Javiera Leniz Martelli
**King's College London**	Jayati Das-Munshi
**King's College London**	Joanna Davies
**King's College London**	Johnny Downs
**King's College London**	Jonathan Hudson
**King's College London**	Katherine Sleeman

Institutions party to the Data Sharing Agreement with NHS England for access to the data in the Secure Data Environment service for England for CVD-COVID-UK/COVID-IMPACT are: Imperial College London, King's College London, London School of Hygiene & Tropical Medicine, Swansea University, University College London, University of Bristol, University of Cambridge, University of Dundee, University of Glasgow, University of Leicester, University of Liverpool, University of Manchester, University of Nottingham, University of Oxford, University of Sheffield, University of Southampton.

CVD-COVID-UK / COVID-IMPACT Consortium Members (11/03/2026)

British Heart Foundation Data Science Centre
Led by Health Data Research UK

Institution	Member Name
King’s College London	Laia Becares
King’s College London	Linglong Qian
King’s College London	Matthew O'Connell
King's College London	Mevhibe Hocaoglu
King’s College London	Natasha Chilman
King's College London	Rachel Cripps
King’s College London	Raquel Catalao
King's College London	Richard Killick
King's College London	Theresa McDonagh
King's College London	Vasa Curcin
King’s College London	Zeljko Kraljevic
London School of Economics & Political Science	Rocco Friebel
London School of Hygiene & Tropical Medicine	Akshay Jagadeesh
London School of Hygiene & Tropical Medicine	Antonio Gasparrini
London School of Hygiene & Tropical Medicine	Arturo de la Cruz
London School of Hygiene & Tropical Medicine	Dorothea Nitsch
London School of Hygiene & Tropical Medicine	Julian Matthewman
London School of Hygiene & Tropical Medicine	Marleen Bokern
London School of Hygiene & Tropical Medicine	Neil Pearce
London School of Hygiene & Tropical Medicine	Patrick Bidulka
London School of Hygiene & Tropical Medicine	Qiuju Li
London School of Hygiene & Tropical Medicine	Sinéad Langan
London School of Hygiene & Tropical Medicine	Thiago Silva
London School of Hygiene & Tropical Medicine	Zhengnan Zhu
Manchester University NHS Foundation Trust	Martin Rutter
Newcastle-upon-Tyne Hospitals NHS Foundation Trust	Alex Grundmann
NHS England	Adam Hollings
NHS England	Angeliki Antonarou
NHS England	Daniel Schofield
NHS England	Deborah Lowe
NHS England	Elizabeth Kelly
NHS England	Richardson
NHS England	Humaira Hussein

Institution	Member Name
NHS England	Jake Kasan
NHS England	Nickie Wareing
NHS England	Russell Healey
NHS England	Shoaib Ali Ajaib
NHS Lanarkshire	Mark Barber
NHS Scotland	Carole Morris
NICE	Felix Greaves
NICE	Jennifer Beveridge
NICE	Seamus Kent
NICE	Thomas Lawrence
NICE	Vandana Ayyar-Gupta
Office for National Statistics	Vahé Nafilyan
Queen Mary University of London	Deepti Gurdasani
Queen's University Belfast	Frank Kee
Royal Brompton and Harefield Hospitals	Paz Tayal
Royal College of Surgeons of England	David Cromwell
Royal Free London NHS Foundation Trust	Amar Shah
Royal Free London NHS Foundation Trust	Swapna Mandal
Royal Papworth Hospital NHS Foundation Trust	Florian Falter
Royal Papworth Hospital NHS Foundation Trust	Joseph Newman
Royal United Hospitals Bath NHS Foundation Trust	Jennifer Rossdale
St George’s University of London	Baboucarr Njie
St George’s University of London	Elijah Behr
St George’s University of London	Nuria Sanchez
St George’s University of London	Rosie Freer
St George’s University of London	Xinkai Wang
Swansea Bay University Health Board	Daniel Harris
Swansea University	Amanda Marchant
Swansea University	Ashley Akbari
Swansea University	Daniel King
Swansea University	David Powell
Swansea University	Elizabeth A Ellins

Institution	Member Name
Swansea University	Gareth Davies
Swansea University	Hoda Abbasizanjani
Swansea University	Huw Strafford
Swansea University	Jane Lyons
Swansea University	Julian Halcox
Swansea University	Laura North
Swansea University	Marcos del Pozo Banos
Swansea University	Owen Pickrell
Swansea University	Ronan Lyons
Swansea University - DATAMIND	Ann John
University College	Robert Aldridge
University College London	Abraham Olvera-Barrios
University College London	Adnan Tufail
University College London	Alasdair Warwick
University College London	Alex Handy
University College London	Alexei Yavlinsky
University College London	Alvina Lai
University College London	Ami Banerjee
University College London	Ana Torralbo
University College London	Ana-Catarina Pinho-Gomes
University College London	Andrej Ivanovic
University College London	Andrew Lambarth
University College London	Anthony Khawaja
University College London	Ashkan Dashtban
University College London	Ashley Dickson
University College London	Becky White
University College London	Christina Pagel
University College London	Christopher Tomlinson
University College London	Chu Siyu
University College London	David Selby
University College London	Eloise Withnell
University College London	Emma Whitfield

Institutions party to the Data Sharing Agreement with NHS England for access to the data in the Secure Data Environment service for England for CVD-COVID-UK/COVID-IMPACT are: Imperial College London, King's College London, London School of Hygiene & Tropical Medicine, Swansea University, University College London, University of Bristol, University of Cambridge, University of Dundee, University of Glasgow, University of Leicester, University of Liverpool, University of Manchester, University of Nottingham, University of Oxford, University of Sheffield, University of Southampton.

CVD-COVID-UK / COVID-IMPACT Consortium Members (11/03/2026)

Institution	Member Name
**University College London**	Evaleen Malgapo
**University College London**	Ferran Espuny-Pujol
**University College London**	Flavien Hardy
**University College London**	Floriaan Schmidt
**University College London**	Freya Allery
**University College London**	Harry Hemingway
**University College London**	Hiroyuki Yoshimura
**University College London**	Honghan Wu
**University College London**	Jinge Wu
**University College London**	Johan Thygesen
**University College London**	Johannes Heyl
**University College London**	Julia Ive
**University College London**	Kate Cheema
**University College London**	Katie Harron
**University College London**	Ken Li
**University College London**	Kerrie Stevenson
**University College London**	Laura Pasea
**University College London**	Louise Choo
**University College London**	Luca Grieco
**University College London**	Manuel Gomes
**University College London**	Mengyun Liu
**University College London**	Michalis Katsoulis
**University College London**	Mohamed Mohamed
**University College London**	Naomi Launders
**University College London**	Nushrat Khan
**University College London**	Paula Lorgelly
**University College London**	Pedro Machado
**University College London**	Pia Hardelid
**University College London**	Qi Huang
**University College London**	Riyaz Patel
**University College London**	Roy Schwartz
**University College London**	Rui Providencia

**Swansea University**	Fatemeh Torabi

Institution	Member Name
**University College London**	Samuel Kim
**University College London**	Scott Isherwood
**University College London**	Simon Ellershaw
**University College London**	Sonya Crowe
**University College London**	Spiros Denaxas
**University College London**	Stephanie Wu
**University College London**	Tuankasfee Hama
**University College London**	Waty Lilaonitkul
**University College London**	Wojtek Banas
**University College London**	Yi Mu
**University College London**	Yohhei Hamada
**University College London**	Yoryos Lyratzopoulos
**University College London / DATAMIND**	David Osborn
**University Hospital of North Midlands**	Arun Pherwani
**University of Aberdeen**	Mary Joan Macleod
**University of Birmingham**	Sarah Wang
**University of Birmingham and Queen Elizabeth Hospital Birmingham**	Mark Thomas
**University of Bradford**	Alejandro Gonzalez-Aquines
**University of Bristol**	Arun Karthikeyan Suseeladevi
**University of Bristol**	Ben Gibbison
**University of Bristol**	Daniel Fudulu
**University of Bristol**	Dann Mitchell
**University of Bristol**	Deborah Lawler
**University of Bristol**	Eleanor Walsh
**University of Bristol**	Elsie Horne
**University of Bristol**	Elvis Annan
**University of Bristol**	Ewan Walker
**University of Bristol**	Falinyi Samson
**University of Bristol**	Gianni Angelini
**University of Bristol**	Jeremy Chan
**University of Bristol**	John Macleod
**University of Bristol**	Jonathan Sterne

Institution	Member Name
**University of Bristol**	Joseph Okeke
**University of Bristol**	Katharine Looker
**University of Bristol**	Kurt Taylor
**University of Bristol**	Livia Pierotti
**University of Bristol**	Luisa Zuccolo
**University of Bristol**	Martha Elwenspoek
**University of Bristol**	Marwa Al Arab
**University of Bristol**	Massimo Caputo
**University of Bristol**	Mercy Nthiwa
**University of Bristol**	Mira Hidajat
**University of Bristol**	Neil Davies
**University of Bristol**	Paul Madley-Dowd
**University of Bristol**	Rachel Denholm
**University of Bristol**	Rochelle Knight
**University of Bristol**	Rupert Payne
**University of Bristol**	Shubhra Sinha
**University of Bristol**	Tim Dong
**University of Bristol**	Tom Palmer
**University of Bristol**	Venexia Walker
**University of Bristol**	Yueying Li
**University of Bristol**	Yvonne Nartey
**University of Cambridge**	Alexia Sampri
**University of Cambridge**	Angela Wood
**University of Cambridge**	Carmen Petitjean
**University of Cambridge**	Chimweta Chilala
**University of Cambridge**	Chriselda Oliver
**University of Cambridge**	David Brind
**University of Cambridge**	Elena Raffetti
**University of Cambridge**	Elias Allara
**University of Cambridge**	Emanuele Di Angelantonio
**University of Cambridge**	Eoin McKinney
**University of Cambridge**	Eric Harshfield

Institutions party to the Data Sharing Agreement with NHS England for access to the data in the Secure Data Environment service for England for CVD-COVID-UK/COVID-IMPACT are: Imperial College London, King's College London, London School of Hygiene & Tropical Medicine, Swansea University, University College London, University of Bristol, University of Cambridge, University of Dundee, University of Glasgow, University of Leicester, University of Liverpool, University of Manchester, University of Nottingham, University of Oxford, University of Sheffield, University of Southampton.

CVD-COVID-UK / COVID-IMPACT Consortium Members (11/03/2026)

British Heart Foundation Data Science Centre
Led by Health Data Research UK

University College London	Ruth Gilbert

Institution	Member Name
**University of Cambridge**	Genevieve Cezard
**University of Cambridge**	Hannah Harrison
**University of Cambridge**	Haoting Zhang
**University of Cambridge**	Holly Pavey
**University of Cambridge**	Isabel Walter
**University of Cambridge**	Jasmine Kiley
**University of Cambridge**	Jessica Barrett
**University of Cambridge**	John Danesh
**University of Cambridge**	John Ford
**University of Cambridge**	Katie Saunders
**University of Cambridge**	Lisa Pennells
**University of Cambridge**	Lois Kim
**University of Cambridge**	Matilda Pitt
**University of Cambridge**	Mayank Dalakoti
**University of Cambridge**	Megan Ritson
**University of Cambridge**	Mike Inouye
**University of Cambridge**	Millie Zhou
**University of Cambridge**	Pearl Liu
**University of Cambridge**	Robert Fletcher
**University of Cambridge**	Rutendo Mapeta
**University of Cambridge**	Samantha Ip
**University of Cambridge**	Spencer Keene
**University of Cambridge**	Stelios Boulitsakis Logothetis
**University of Cambridge**	Stephen Kaptoge
**University of Cambridge**	Tianxiao Wang
**University of Cambridge**	Tom Pape
**University of Cambridge**	Wen Shi
**University of Cambridge**	Xilin Jiang
**University of Cambridge**	Xiyun Jiang
**University of Cambridge**	Yanfan Li
**University of Dundee**	Daniel Morales
**University of Dundee**	Huan Wang

Institution	Member Name
**University of Dundee**	Samira Bell
**University of Edinburgh**	Alan Carson
**University of Edinburgh**	Alice Hosking
**University of Edinburgh**	Annemarie Docherty
**University of Edinburgh**	Athina Spiliopoulou
**University of Edinburgh**	Baljean Dhillon
**University of Edinburgh**	Carlos Sanchez Soriano
**University of Edinburgh**	Caroline Jackson
**University of Edinburgh**	Christian Schnier
**University of Edinburgh**	Claire Tochel
**University of Edinburgh**	Dave Yeung
**University of Edinburgh**	Gavin Chapman
**University of Edinburgh**	Gwenetta Curry
**University of Edinburgh**	Helen Colhoun
**University of Edinburgh**	Joe Mellor
**University of Edinburgh**	Kelly Fleetwood
**University of Edinburgh**	Laura Sherlock
**University of Edinburgh**	Luke Blackbourn
**University of Edinburgh**	Michelle Williams
**University of Edinburgh**	Miguel Bernabeu Llinares
**University of Edinburgh**	Niamh McLennan
**University of Edinburgh**	Rebecca Reynolds
**University of Edinburgh**	Richard Chin
**University of Edinburgh**	Steven Kerr
**University of Edinburgh**	Tim Wilkinson
**University of Edinburgh**	Verónica Cabreira
**University of Edinburgh**	William Berthon
**University of Edinburgh**	William Whiteley
**University of Exeter**	John Dennis
**University of Exeter**	Kerry Pearn
**University of Exeter**	Michael Allen
**University of Glasgow**	Angela Henderson

University of Cambridge	Fabian Falck

Institution	Member Name
**University of Glasgow**	Colin Berry
**University of Glasgow**	Craig Melville
**University of Glasgow**	Dennis Tran
**University of Glasgow**	Filip Sosenko
**University of Glasgow**	Frederick Ho
**University of Glasgow**	Jill Pell
**University of Glasgow**	Jocelyn Friday
**University of Glasgow**	John Cleland
**University of Glasgow**	Naveed Sattar
**University of Glasgow**	Ninian Lang
**University of Glasgow**	Salil Deo
**University of Glasgow**	Sandosh Padmanabhan
**University of Glasgow**	Terry Quinn
**University of Leeds**	Charlotte Sturley
**University of Leeds**	Jianhua Wu
**University of Leeds**	Jonathan Batty
**University of Leeds**	Marlous Hall
**University of Leeds**	Ramesh Nadarajah
**University of Leeds**	Tamara del Toro
**University of Leicester**	Anna Hansell
**University of Leicester**	Anvesha Singh
**University of Leicester**	Cameron Razieh
**University of Leicester**	Claire Lawson
**University of Leicester**	Clare Gillies
**University of Leicester**	Elpida Vounzoulaki
**University of Leicester**	Francesco Zaccardi
**University of Leicester**	Iain Squire
**University of Leicester**	Kamlesh Khunti
**University of Leicester**	Matthew Bown
**University of Leicester**	Muhammad Rashid
**University of Leicester**	Sharmin Shabnam
**University of Leicester**	Shirley Sze

Institutions party to the Data Sharing Agreement with NHS England for access to the data in the Secure Data Environment service for England for CVD-COVID-UK/COVID-IMPACT are: Imperial College London, King's College London, London School of Hygiene & Tropical Medicine, Swansea University, University College London, University of Bristol, University of Cambridge, University of Dundee, University of Glasgow, University of Leicester, University of Liverpool, University of Manchester, University of Nottingham, University of Oxford, University of Sheffield, University of Southampton.

CVD-COVID-UK / COVID-IMPACT Consortium Members (11/03/2026)

British Heart Foundation Data Science Centre
Led by Health Data Research UK

Institution	Member Name
University of Dundee	Ify Mordi
University of Glasgow	Clea du Toit
University of Leicester	Tom Yates

Institution	Member Name
University of Leicester	Yogini Chudasama
University of Lisbon	João Malato
University of Liverpool	Andrew Mason
University of Liverpool	Benedict Michael
University of Liverpool	Caroline Dale
University of Liverpool	David Hughes
University of Liverpool	Francesca Zaccagnino
University of Liverpool	Giulia Ballabio
University of Liverpool	Ike Chukwudi
University of Liverpool	Maria Sudell
University of Liverpool	Mark Green
University of Liverpool	Munir Pirmohamed
University of Liverpool	Orla Hilton
University of Liverpool	Pardis Biglarbeigi
University of Liverpool	Reecha Sofat
University of Liverpool	Rohan Takhar
University of Liverpool	Ruwanthi Kolamunnage-Dona
University of Liverpool	Stephen McKeever
University of Manchester	Bernard Keavney
University of Manchester	Catriona Harrison
University of Manchester	Craig Smith
University of Manchester	David Jenkins
University of Manchester	Eva Henning
University of Manchester	Evan Kontopantelis
University of Manchester	George Tilston
University of Manchester	Glen Martin
University of Manchester	Hector Chinoy
University of Manchester	Joseph Firth
University of Manchester	Lana Bojanić
University of Manchester	Matthew Sperrin
University of Manchester	Max Lyon
University of Manchester	Ruth Norris
University of Manchester	Ruth Watkinson
University of Manchester	Sarah Steeg
University of Manchester	Simon Frain
University of Manchester	Simon Williams
University of Manchester	Steven Zhao
University of Manchester	Zenas Yiu
University of Milan	Federico Fama
University of Milan	Marta Colaneri
University of Newcastle	Camille Carroll
University of Newcastle	Charlotte Parbery-Clark
University of Newcastle	Dexter Canoy
University of Newcastle	Precious Onyeachu
University of Nottingham	Fiona Pearce
University of Nottingham	Laila Tata
University of Nottingham	Ralph Akyea
University of Nottingham	Stephanie Lax
University of Oxford	Aashna Uppal
University of Oxford	Akshay Shah
University of Oxford	Antonella Delmestri
University of Oxford	Antony Palmer
University of Oxford	Ben Goldacre
University of Oxford	Ben Lacey
University of Oxford	Dani Prieto-Alhambra
University of Oxford	Eva Morris
University of Oxford	Gardash Bakhishli
University of Oxford	George Nicholson
University of Oxford	Hayley Evans
University of Oxford	James Sheppard
University of Oxford	Joseph Kamtchum Tatuene
University of Oxford	Julia Hippisley-Cox
University of Oxford	Lucy Wright
University of Oxford	Marta Pineda Moncusi
University of Oxford	Mohammad Mamouei
University of Oxford	Nick Hall
University of Oxford	Parag Gajendragadkar
University of Oxford	Paula Dhiman
University of Oxford	Qingze Gu
University of Oxford	Ramla Khan
University of Oxford	Raph Goldacre
University of Oxford	Richard Williams
University of Oxford	Salma Chaudhry
University of Oxford	Sara Khalid
University of Oxford	Seb Bacon
University of Oxford	Seyed Alireza Hasheminasab
University of Oxford	Shishir Rao
University of Oxford	Xiaomin Zhong
University of Oxford	Zeinab Bidel Taleshmekaeil
University of Oxford/KU Leuven	Nathalie Conrad
University of Plymouth	Marie-Louise Zeissler
University of Sheffield	Norman Briffa
University of Sheffield	Simone Croft
University of Sheffield	Suzanne Mason
University of Sheffield	Tim Chico
University of Southampton	Nazrul Islam
University of Strathclyde	Amanj Kurdi
University of Strathclyde	Kim Kavanagh
University of Strathclyde	Marion Bennie
University of Strathlcyde	Tanja Mueller
University of Warwick	Harry Wilde
University of Warwick	Majel McGranahan
University of York	Christina van der Feltz-Cornelis

Institutions party to the Data Sharing Agreement with NHS England for access to the data in the Secure Data Environment service for England for CVD-COVID-UK/COVID-IMPACT are: Imperial College London, King's College London, London School of Hygiene & Tropical Medicine, Swansea University, University College London, University of Bristol, University of Cambridge, University of Dundee, University of Glasgow, University of Leicester, University of Liverpool, University of Manchester, University of Nottingham, University of Oxford, University of Sheffield, University of Southampton.

CVD-COVID-UK / COVID-IMPACT Consortium Members (11/03/2026)

British Heart Foundation Data Science Centre
Led by Health Data Research UK

**University of Manchester**	Maya Buch
**University of Manchester**	Richard Williams

**University of Oxford**	Kazem Rahimi
**University of Oxford**	Linxin Li

**University of York**	Han-I Wang
**University of York**	Lorna Fraser

Institution	Member Name
**Wellcome Sanger Institute**	Tapiwa Tungamirai

Institutions party to the Data Sharing Agreement with NHS England for access to the data in the Secure Data Environment service for England for CVD-COVID-UK/COVID-IMPACT are: Imperial College London, King's College London, London School of Hygiene & Tropical Medicine, Swansea University, University College London, University of Bristol, University of Cambridge, University of Dundee, University of Glasgow, University of Leicester, University of Liverpool, University of Manchester, University of Nottingham, University of Oxford, University of Sheffield, University of Southampton.
